# Screening for Fall Risks in the Emergency Department: A Novel Nursing-Driven Program

**DOI:** 10.5811/westjem.2015.10.26097

**Published:** 2015-12-10

**Authors:** Jill M. Huded, Scott M. Dresden, Stephanie J. Gravenor, Theresa Rowe, Lee A. Lindquist

**Affiliations:** * Case Western Reserve University School of Medicine, Department of Medicine, Cleveland, Ohio; † Louis Stokes VA Medical Center, Department of Medicine, Cleveland, Ohio; ‡ Northwestern University Feinberg School of Medicine, Department of Emergency Medicine, Chicago, Illinois; § Northwestern University Feinberg School of Medicine, Department of Medicine, Division of General Internal Medicine and Geriatrics, Chicago, Illinois

## Abstract

**Introduction:**

Seniors represent the fasting growing population in the U.S., accounting for 20.3 million visits to emergency departments (EDs) annually. The ED visit can provide an opportunity for identifying seniors at high risk of falls. We sought to incorporate the Timed Up & Go Test (TUGT), a commonly used falls screening tool, into the ED encounter to identify seniors at high fall risk and prompt interventions through a geriatric nurse liaison (GNL) model.

**Methods:**

Patients aged 65 and older presenting to an urban ED were evaluated by a team of ED nurses trained in care coordination and geriatric assessment skills. They performed fall risk screening with the TUGT. Patients with abnormal TUGT results could then be referred to physical therapy (PT), social work or home health as determined by the GNL.

**Results:**

Gait assessment with the TUGT was performed on 443 elderly patients between 4/1/13 and 5/31/14. A prior fall was reported in 37% of patients in the previous six months. Of those screened with the TUGT, 368 patients experienced a positive result. Interventions for positive results included ED-based PT (n=63, 17.1%), outpatient PT referrals (n=56, 12.2%) and social work consultation (n=162, 44%).

**Conclusion:**

The ED visit may provide an opportunity for older adults to be screened for fall risk. Our results show ED nurses can conduct the TUGT, a validated and time efficient screen, and place appropriate referrals based on assessment results. Identifying and intervening on high fall risk patients who visit the ED has the potential to improve the trajectory of functional decline in our elderly population.

## INTRODUCTION

Seniors over the age of 65 years represent the fastest growing population in the U.S., accounting for 20.3 million visits to emergency departments (EDs) annually. 1 Over two million (10%) of these visits are preceded by a nonfatal fall, 2 highlighting the mounting problem of impaired mobility among this population. Approximately one third of individuals over the age of 65 will fall at least once yearly, leading to fractures, surgery, inpatient admissions, prolonged rehabilitation and death. 3

The ED visit may be a sentinel event to prevent such falls and change the trajectory of older adults’ functional decline. Identifying a senior at high risk of falls can lead to targeted therapies through pharmacy, social work, home health, and physical therapy (PT) referrals. In preparing for discharge from the ED or hospital, these teams can promote individualized and multi-component exercise programs, the most effective strategy in reducing falls among community-dwelling elderly. 4 Despite a recommendation for ED fall risk screening by national emergency medicine societies, there are very few validated screening protocols specific to the ED. 5 A multidisciplinary approach including detailed medical and occupation therapy evaluations for those presenting to the ED after a fall has previously been described with effective results. 6 Less is known about such assessments for those seen in the ED who have not immediately fallen but are at high risk for falls, and for those receiving PT evaluations in the ED.

One tool used to identify risk of falling is the Timed Up and Go Test (TUGT). This validated assessment of gait and balance is quick, freely available, and requires minimal training and supplies (e.g. chair and timer). 7 To perform the TUGT, the patient’s ability to rise from a seated position, walk three meters, turn, walk back and sit down is observed and timed. The TUGT is designed to predict occurrence of falls, as individuals 65 years and older with a TUGT of 12.4 seconds or greater are three times more likely to fall in the next year. 8, 9 The purpose of this study is to describe the use of TUGT assessments performed by geriatric nurses in the ED and nurse initiated interventions for positive TUGTs.

## METHODS

### Setting

This study was conducted as part of the Geriatric ED Innovations through Workforce, Informatics, and Structural Enhancements (GEDI WISE) program. GEDI WISE is three-site initiative funded by the Centers for Medicare and Medicaid Services to improve the care of older patients in the ED. The study site was the ED of an urban, academic, Level 1 trauma center with 56 beds. The study site has an annual volume over 80,000 patients and an annual geriatric volume of over 16,000 patients, with an overall admission rate of 32% (inpatient and observation) and a baseline geriatric admission rate of 60% (inpatient and observation) prior to the implementation of GEDI WISE. Prospective data was collected on adults ≥65 years who had a geriatric nurse liaison (GNL) assessment between April 1, 2013 and May, 31, 2014.

### GNL Assessments

As training for this initiative, GNLs participated in a multidisciplinary curriculum developed by emergency medicine and geriatric educational experts. In the ED, GNLs perform assessments of cognition, delirium, functional status, caregiver strain and gait for geriatric patients presenting to the ED through a previously described protocol. 10 TUGT was performed on patients at the discretion of GNLs and emergency physicians. GNLs were encouraged to assess patients with a recent fall, concern for gait, balance or strength impairment, or need for a mobility device. Those with unstable vital signs, pending or abnormal head imaging, acute fractures, or altered mental status did not receive a TUGT evaluation. Through a collaborative discussion with the GNL and the patient’s clinical team (nurse, resident, or attending) a joint decision was made as to which fall risk intervention was most appropriate and should be pursued while the patient resided in the ED. Abnormal TUGT was defined as greater than 12 seconds, indicating high fall risk as previously described in the literature.^8^,^9^

### Data Analysis

Approval for this project was granted by the institutional review board. Demographic data, mean TUGT and GNL-initiated therapeutic interventions performed during the ED visit were abstracted by review of the electronic medical record and Enterprise Data Warehouse. An abnormal TUGT or any concerns for gait or balance by direct observation could result in a therapeutic intervention. Interventions were categorized into PT, social work, Department on Aging referrals, and discussions with primary care physicians, family or caregivers. PT interventions included PT consults in the ED, PT consults during an anticipated observation or inpatient stay, home PT orders through home health, or outpatient PT orders.

## RESULTS

During the study period, 19,511 geriatric patients were treated in the ED, and 1,135 (5.8%) were evaluated by a GNL. Of patients evaluated by a GNL, TUGT was performed on 443 (39.0%) patients. The average age of patients undergoing TUGT was 79.8 years, with 37% reporting a fall in the preceding12 months. Among those undergoing the TUGT, the documented chief complaint included “fall” or “fell” for 70 (15.8%) patients. Three hundred thirteen (70.7%) of patients evaluated with the TUGT were discharged from the ED, 65 (14.5%) were admitted as inpatients and 65 (14.5%) were admitted under observation status. High independence as measured by Katz Activity of Daily Living was seen in 56% (n=249). No cognitive impairment was found in 82% (n=363), and 7% (n=31) had moderate to severe cognitive deficits as measured by the Short Portable Mental Status Questionnaire (Table 1).

We saw positive TUGT scores in 368 of the 443 (83%) patients undergoing gait evaluation (i.e. required longer than 12 seconds to complete the three meter walk). Seventeen percent (n=75) had normal TUGT scores, 27% (n=120) required between 12.1 and 19 seconds, 36% (n=158) required between 20 and 32.6 seconds, and 20% (n=90) took longer than 32.6 seconds to complete the test. Assistive mobility devices were used at baseline in 201 of the 368 (55%) patients with positive TUGT scores.

PT consults were performed in the ED for 17.1% of patients with positive TUGT scores. For patients directly discharged home from the ED 12.2% were provided with a script for outpatient PT. Social work consults were completed in the ED for 44% of cases, and primary care providers were contacted by the GEDI team to notify them of a patient’s ED evaluation or concern for gait instability in 7% of cases. For those with positive TUGT scores, 74% were discharged home (n=274) and the remainder were admitted under inpatient or observation status (Table 2).

## DISCUSSION

The rising number of elderly patients in the ED presents opportunities to identify those at high risk of falls, refer for a more in-depth mobility assessment with PT, and initiate mobility care plans. In this study describing the implementation of a falls-identification program in the ED, the TUGT test was successfully incorporated into the routine care of geriatric patients presenting for acute care evaluation. Though this study was performed with the support of a CMS award, a low-cost protocol is feasible. The TUGT is a formalized way of measuring how fast a patient walks. This test can be performed by a bedside nurse, or patient care technician, and does not necessarily require a dedicated GNL. The key to the protocol is having a plan for prolonged TUGT scores. For patients who are felt to be safe for discharge, outpatient PT is a reasonable option (often in conjunction with the patient’s primary care physician). For patients who are to be admitted, inpatient PT would be appropriate. Similar screenings performed in EDs by patient care technicians for cognitive impairment, fall risk and functional decline have previously been incorporated into the work flow of a Level I trauma center ED where fewer than 25% of physicians routinely screened for geriatric syndromes. 11 The use of such personnel adds valuable information and provides insight into a patient’s current functional status to stratify them into fall risk categories. Appropriate referrals to PT, both in the hospital and upon discharge, and discussion with social work, primary care providers and caregivers can be initiated.

Over 70% of our population was discharged home from the ED, many with slow TUGT scores, who would benefit from PT in this immediate sub-acute setting. Only 17% of patients with positive TUGT scores received a PT consult in the ED, and 20% received either an order for home or outpatient PT. PT consults in the ED were deferred to inpatient therapists if a patient was certain to require admission. Home or outpatient PT orders were not ordered if a patient was at their baseline functional status, they were undergoing therapy prior to presentation, or acuity of addressing other medical concerns took precedence. Further research is needed to determine if 12 seconds is an appropriate cutoff for ED patients, if any TUGT cutoff represents a requirement for safe discharge home, and if PT consultation provides a benefit to patients with prolonged TUGT scores. Additionally, optimal communication about fall risk needs across transitions of care should be developed.

## LIMITATIONS

This is the first study showing that a protocoled method of identifying fall risk in elderly patients is possible for those presenting to the ED for acute care needs other than a recent fall. Several limitations deserve mention. This was a single-site study and was incorporated into a geriatric-specific protocol supported by specialized registered nurse (RN) staff already in place. All RNs performing the TUGT were initially trained as emergency medicine nurses and continued to have weekly ED shifts working in a traditional RN capacity. The TUGT is designed to be a simple test that all health personnel can perform. EDs initiating similar screening programs may need to invest more energy in ensuring appropriate interventions for positive TUGT scores than the actual training of TUGT administrators.

We recognize that the TUGT is one screen in addition to many already being emphasized in the ED; however, targeting appropriate older patients may minimize the workload and is timely in light of geriatric-specific EDs evolving across the U.S. While a small percentage of the potentially eligible geriatric patients were screened with the TUGT, we believe the sample of patients who were assessed by GNLs represents a high-risk population, as identified by GEDI WISE protocol, or clinician consult; 15.8% of screened patients presented to the ED after a fall, and this may have increased the perceived benefit of the TUGT screen compared to a more widespread screening protocol. However we believe this high rate of previous fall in the screened population demonstrates appropriate targeting of screening to a population at high risk for repeat falls. Without intervention more that 20% will present to the ED within 12 months with another fall-related diagnosis. 12 Finally, previously defined TUGT cutoffs for outpatients may not be the most appropriate cutoffs for older adults in the ED who are presenting with acute medical conditions that may affect their gait.

## CONCLUSION

In a healthcare system with rising numbers of geriatric patients seeking care in the ED, using available tools to identify patients at risk for debilitating, even fatal, falls before they occur is important for patient safety and functional independence. When at-risk patients are identified, interventions can be implemented to prevent future falls. With resources available in the ED such as nurses, social workers, physical therapists, and patient care technicians, the ED can serve as a key source of identifying seniors at risk of falls. Fall-risk identification with simple, validated tests, such as the TUGT and ED-based interventions, are important to change the trajectory of functional decline in our elderly population.

## Figures and Tables

**Table 1:** Participant characteristics (n=443).

Characteristic	n	%
Age, y (SD)	79.8	(±7.9)
Female	277	(62.5)
Race		
White	237	(53.5)
Black	157	(35.4)
Hispanic	30	(6.8)
Asian	13	(2.9)
Other or unknown	6	(1.4)
Cognition (SPMSQ)		
Normal	363	(82.0)
Mild impairment	46	(10.4)
Moderate or severe impairment	31	(7.0)
Could not assess	3	(0.7)
Functional status (Katz ADLs)		
Low independence	5	(1.1)
Moderate independence	137	(30.9)
High independence	249	(56.2)
Could not assess	52	(11.7)
Fall in last 12 months	165	(37.2)

*SD,* standard deviation; *SPMSQ,* short portable mental status questionnaire; *ADLs,* activities of daily living

**Table 2:** Emergency department (ED) interventions for positive timed up and go test (n=368).

Intervention	n	%
PT order in ED	63	(17.1)
Outpatient PT referral, ordered from ED	56	(12.2)
Home PT referral, ordered from ED	30	(8.2)
Social work referral in ED	162	(44)
Discussed with family or caregiver	75	(20.4)
Discussed with PCP	27	(7.3)
Department of aging referral	40	(10.9)
Discharged home from ED	274	(74.5)
Admitted to inpatient status	44	(12.0)
Placed in observation status	50	(13.6)

*PT,* physical therapy; *PCP,* primary care provider
